# Correlation of age and skeletal effects after miniscrew assisted rapid palatal expansion 

**DOI:** 10.4317/jced.60211

**Published:** 2023-04-01

**Authors:** Cecilia-Maria Marín, Maria del Carmen Benitez, Aldo Otazu, Dino-Marcelo Torres, Paula Cotrin, Celia-Regina-Maio Pinzan-Vercelino, Fabricio-Pinelli Valarelli, Karina-Maria-Salvatore Freitas

**Affiliations:** 1 DDS, MSc. Orthodontic graduate student. Ingá University Center UNINGA. Maringá, Brazil; 2 DDS, MSc. Ph.D. Professor. Advanced Dentistry Institute IOA. Asunción, Paraguay; 3 DDS, MSc. Professor. Ingá University Center UNINGA. Maringá, Brazil

## Abstract

**Background:**

To evaluate the skeletal and dentoalveolar effects after miniscrew assisted rapid palatal expansion (MARPE) and their correlation with the age of the patients. Settings and sample population: Sample comprised 19 patients with maxillary atresia and posterior crossbite, treated with MARPE. Cone-beam computed tomographs (CBCT) were evaluated before and after expansion. Three patients were excluded since the midpalatal suture was not opened. Thus, 16 patients (11 female; 5 male) were evaluated, with a mean age of 24.92 years (s.d.=7.60). The time between the installation of MARPE and the second CBCT was, on average, 1.64 months (s.d.=1.12).

**Material and Methods:**

Linear and angular measurements were performed: bone thickness and level, tooth inclination, transverse dental widths, and nasal base and jugula widths. Comparison was performed with dependent t-test and correlations with Pearson coefficient.

**Results:**

MARPE was 84.2% successful. There was significant reduction in the buccal bone thickness of the first molars and an increase in the palatal bone thickness of all teeth. First molars showed significant buccal inclination. All transverse dimensions showed a significant increase. Older patients tended to show a less maxillary transverse skeletal increase. A greater maxillary transverse increase was accompanied by a greater intermolar width increase and also a greater buccal bone loss in the mesiobuccal roots of the maxillary first molars.

**Conclusions:**

MARPE corrected the maxillary atresia in adult patients, with significant transverse increases, a slight decrease in buccal bone thickness and buccal inclination of the first molars, combining skeletal and dental effects. Older patients presented less transverse skeletal increases.

** Key words:**Palatal expansion technique, skeletal anchorage, cone-beam computed tomography.

## Introduction

Rapid maxillary expansion (RME) has been extensively used in Orthodontics to increase the maxillary transverse dimensions in growing patients (1,2).

As age progresses, the suture is usually obliterated by calcified tissue (3). In adults, it is recommended that RME be done surgically with medial and pterygomaxillary palatal osteotomies to provide a significant maxillary expansion with an increase in arch perimeter (4,5). This treatment modality helps to overcome the increased resistance of the bone suture in adults (6). Surgically assisted rapid maxillary expansion (SARME) can present complications, it has a high cost and complex treatment process (6,7).

Recent evidence indicates that RME can be performed effectively without the need for surgery in young adults using palatal skeletal anchorage (8,9). The appliance used for Mini-implant Assisted Rapid Palatal Expansion (MARPE) is a modification of the conventional appliance used for RME. Installation of mini-implants is a simple, effective and stable procedure that can avoid the need for surgical procedures in young adult patients with transverse maxillary deficiency (10-12).

RME use teeth as anchorage and presents immediate effects as buccal inclination, reduction of buccal bone thickness and level of the posterior teeth (13-15). The main functional difference in MARPE is that mini-implants are placed on the palate so the forces are directed to the palate and can guarantee the expansion of the basal bone minimizing dentoalveolar inclination and deleterious dental effects (16).

Choi *et al*. (10) evaluated the long-term stability of MARPE in young adults with transverse maxillary deficiency. The midpalatal suture opened in a triangular shape; the smallest increase was observed in the width of the nasal cavity (1.07mm) and the largest increase in the intermolar width (8.32mm). The increase in the intermolar distance was 3.94 times greater than the increase of the maxilla (2.11mm). The clinical crown height and the amount of gingival recession were not significant and treatment was considered clinically accepTable and sTable. MARPE’s success rate was 86.96% (10).

Lagravère *et al*. (17) compared the skeletal changes in transverse, vertical and anteroposterior directions and the dental changes using tooth-supported and bone-supported expanders and both appliances showed similar results. The greatest changes were seen in the transverse direction; the vertical and anteroposterior changes were not significant (17).

Lagravère *et al*. (18) sought to determinate the skeletal and dental differences after RME with and without mini-implant support and tooth inclination was observed in the molars for both expansion treatments, while the premolars showed greater inclination with the tooth-supported appliance (18).

Toklu *et al*. (19) observed significant periodontal changes in molars and premolars. In the first molars, there was a decrease in bone thickness in the molars. For the premolars, the buccal bone plate decreased only in the tooth-supported appliance (19). Pham and Lagravère (20) concluded that changes in the alveolar bone level were similar in both RME treatments, tooth and bone-supported.

Some studies show changes in bone thickness and level, dental inclination and transversal dimensions of the maxilla after MARPE. Still, very few in adults and none of them correlate these changes with the age of the patients. This study aimed to evaluate the skeletal and dentoalveolar effects after miniscrew assisted rapid palatal expansion (MARPE) and their correlation with the age of the patients.

## Material and Methods

-Materials 

This study was approved by the Research Ethics Committee of Ingá University Center, Maringá, PR, Brazil (CAAE 04614318.5.0000.5220).

The sample size was calculated based on a 5% alpha and 20% beta to achieve test power of 80%, in a single sample, to detect a mean difference of 0.5mm with a standard deviation of 0.66mm in the mesiobuccal bone thickness of the maxillary first molar (19). Thus, sample size calculation resulted in the need for 16 patients.

This study was performed on patients treated at the Advanced Dentistry Institute, Asuncion, Paraguay, between 2017 and 2019. The patients signed an informed consent to participate in the study.

The following inclusion criteria were considered: patients between 17 and 40 years old with no history of previous orthodontic treatment, complete permanent dentition erupted up to second molars without periodontal disease or cleft palate and with atresia of the maxilla with unilateral or bilateral posterior crossbite.

Thus, 19 patients were selected, with a total of 11 females and 8 males, with a mean initial age of 25.58 years (SD=7.33). Cone-Beam Computed Tomographies (CBCT) were performed before the installation of the expansion appliance (T1) and after MARPE (T2). The time between the installation of MARPE and the second CBCT was, on average, 1.64 months (SD=1.12). MARPE was maintained for at least 4 months after the end of the activations and soon after, the patients were treated with fixed appliances.

**Figure 1:**
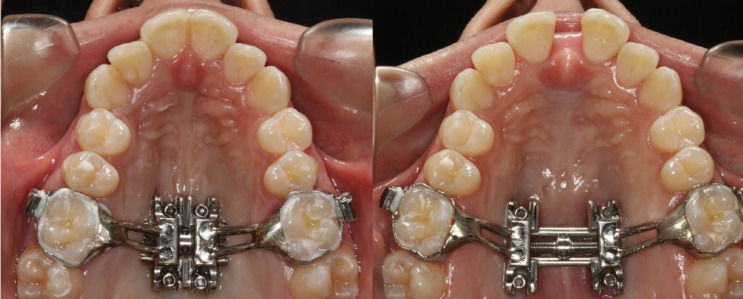
Occlusal views before and after expansion with MARPE.

All treatments were conducted by two professionals, with the same type of MARPE appliance (Peclab, Belo Horizonte, Brazil) with bands in the maxillary first molars and anchored on the palate with four titanium mini-implants of 1.8mm in diameter and 8mm in length. Activations were performed as follows: ¼-turn in the morning and ¼ -turn at night until the palatal cusp of the maxillary first molars touches the buccal cusp of the mandibular first molars. It was clinically observed if there was an interincisive diastema to evaluate the success in opening the midpalatal suture (Fig. 1).

Of the 19 selected patients, only 16 presented interincisive diastema and opening of the midpalatal suture, confirmed by an occlusal radiograph. Their CBCT scans were not included in the measurements to avoid influencing the results.

In the 16 patients who presented opening of the midpalatal suture, the monocortical insertion of the mini-implants of the MARPE occurred in only one of them, in other patient, the two posteriors mini-implant were bicortical inserted and the two anterior, monocortical. The mini-implants of the other patients had bicortical insertion.

Of the 3 patients where there was no opening in the midpalatal suture, one of them presented the bicortical insertion of the posterior mini-implants and the anterior ones monocortical, and the mini-implants of the other two patients were placed bicortical.

Thus, the final sample consisted of 16 patients (11 female; 5 male), with a mean initial age of 24.92 years (SD=7.60).

-Methods 

All CBCT scans were performed using the Orthophos SL 2D/3D device (Sirona Dental Systems GmbH, Germany), with the following image acquisition protocol: field of view (FOV) 8 cm Ø x 5.5 cm high (maxilla) and the 3D resolution of 0.08 mm in HD mode (length in isotropic voxels). The images were saved in DICOM format and the program selected for the measurements was Dolphin 3D version 11.95 Premium (Dolphin Imaging & Management Solutions, Chatsworth, USA). This program allows viewing the images in three planes: sagittal, coronal and axial.

To allow reliable measurements, head position was standardized. In frontal view, the reference plane used passed through the lowest points of the infraorbital foramen; in lateral view, the plane that passes through the anterior and posterior nasal spines and in axial view, the line passing through the galli crest and the center of the foramen magnum.

**Figure 2:**
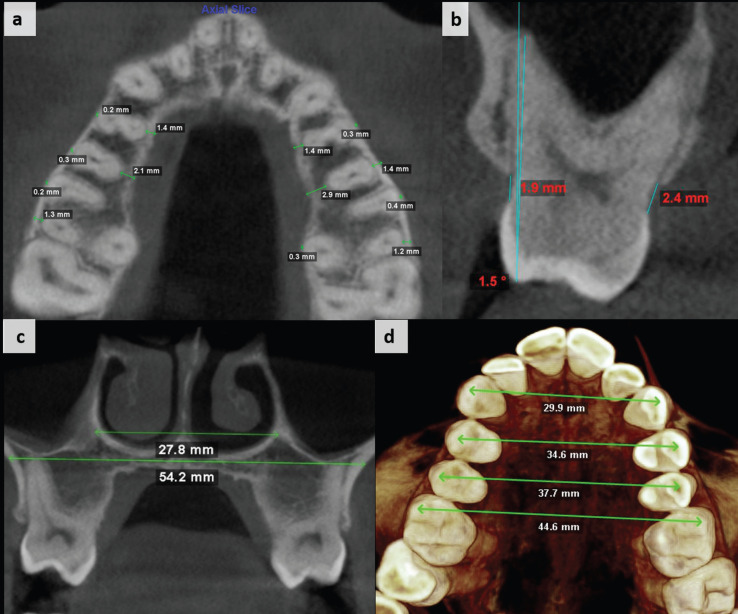
Linear and angular measurements evaluated: a) Buccal and palatal bone thickness; b) Buccal and palatal bone level and tooth inclination; c) Nasal base and jugula widths; and d) Interdental widths.

Linear and angular measurements were made in the maxillary first and second premolars and first molars, as described in Table 1 and Figure 2.

**Table 1:**
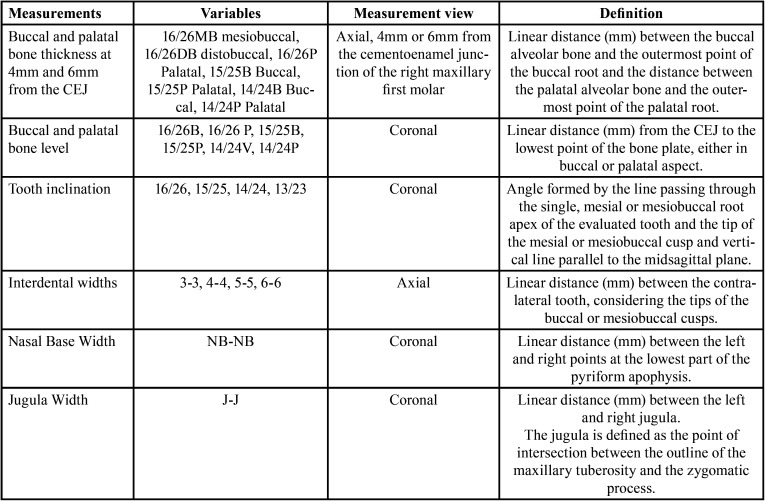
Linear and angular measurements evaluated.

-Error study

The intraexaminer error was assessed by re-measuring, after a month interval, the initial and final CBCT scans of 6 patients randomly selected, totaling 12 CBCT scans. The intraexaminer reliability was assessed using intraclass correlation coefficients (21).

-Statistical analysis

The normality of the data was verified by the Shapiro-Wilk test.

Descriptive statistics of the initial age and time between the installation of MARPE and the second CBCT were performed.

The comparison of the variables between stages before and after MARPE was performed using dependent t test.

The Pearson correlation coefficient was used to evaluate the correlations between skeletal changes with MARPE and the age of the patients and also between the transverse changes, inclination and bone loss of the first molars.

The tests were performed with Statistica software (Statsoft, Tulsa, USA) and data were considered significant for *P*<0.05.

## Results

Intraclass correlation coefficients (ICCs) of the variables varied from 0.91to 0.97, indicating excellent intra-rater agreement (22).

Of the 19 cases treated with MARPE, 16 were successful in opening the midpalatal suture, representing 84.2% of the total. The 3 patients who were not successful were males and aged 23, 31 and 32 years.

There was a significant decrease in buccal bone thickness at 4 mm in the mesiobuccal root of the first molars and the distal root of the left first molar and an increase in the palatal bone thickness of the right first molar, second premolars and right first premolar (Table 2). At 6 mm, there was a statistically significant decrease in the buccal bone thickness of the right first molar and an increase in the palatal bone thickness of the right first molar, second premolars, and first left premolar (Table 2). The bone level was not changed with MARPE (Table 3).

**Table 2:**
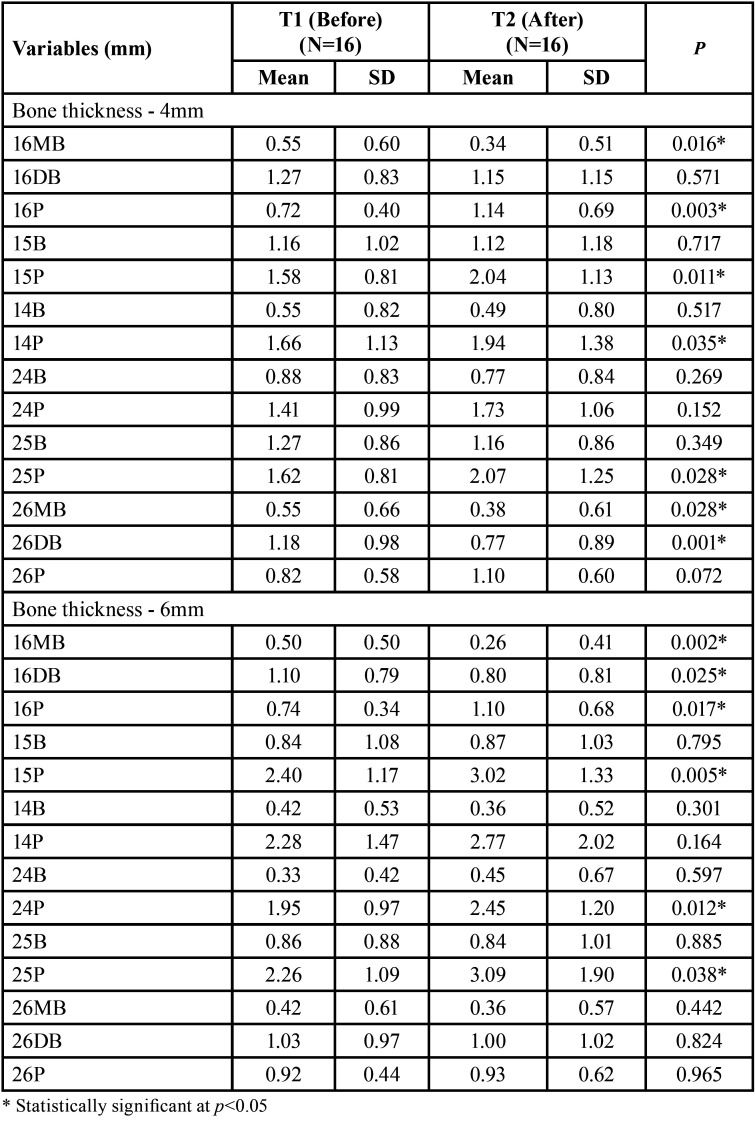
Comparison of bone thickness at 4mm and at 6mm from CEJ before and after MARPE (dependent t test) (N=16).

**Table 3:**
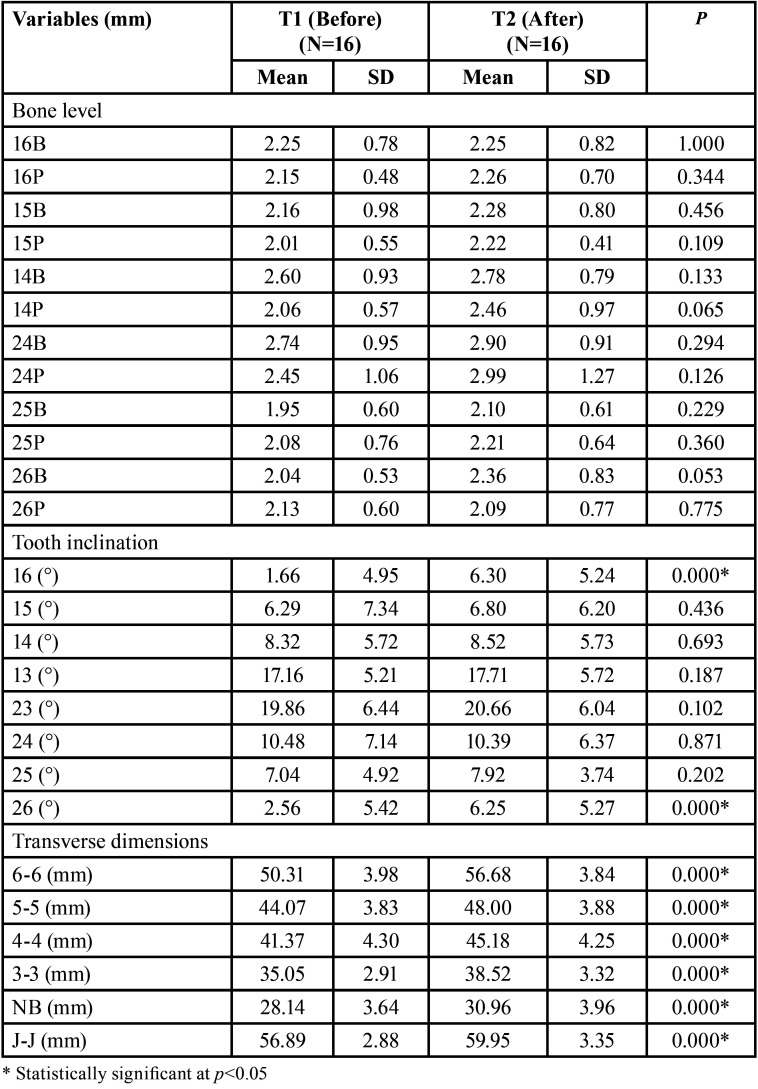
Comparison of bone level, tooth inclination and transverse dimensions before and after MARPE (dependent t test) (N=16).

There was a significant increase in the buccal inclination of the maxillary first molars (Table 3). There was a statistically significant increase in all interdental distances, as well as the nasal base and jugula widths. The midpalatal suture was opened in a triangular shape, with the smallest increase observed in the nasal base (2.82mm) and the largest increase observed in the intermolar distance (6.37mm). Skeletal expansion, measured in the maxillary width (J-J=3.06mm), represented 48% of the total transversal gain measured at the molar level (Table 3).

There was a negative correlation between the age of the patients and the nasal base and jugula width changes, indicating that the greater the age, the smaller the transverse skeletal increase (Table 4). The greater the maxillary transverse skeletal increase (J-J), the greater the nasal base increase and the intermolar width increase, and the greater the buccal bone loss in the mesiobuccal root of the first molars (6-6) (Table 4).

**Table 4:**
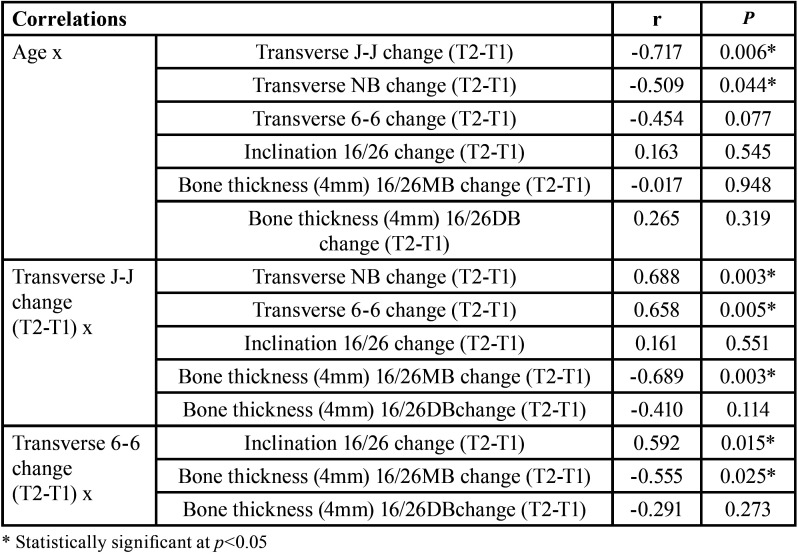
Correlations of the age of the patients and the skeletal and dentoalveolar effects (Pearson’s correlation coefficient).

## Discussion

The sample consisted of young adults with a mean age of 24.92 years. As the success of conventional RME is not guaranteed at this age (13), MARPE was the treatment choice. The opening of the midpalatal suture was observed in 84.2% of the patients (16 of 19), similar to the previous studies (6,10,23). Other authors have achieved 100% success (9,24). Differences in the suture maturation stage can justify the expansion failure in adults (25).

RME performed with the aid of mini-implants or not is achieved through the orthopedic expansion given by the opening of the midpalatal suture and the dental expansion given by the expansion or inclination of the alveolus and the teeth inclination (15). The skeletal effects are similar in both RME and MARPE, according to previous studies (17,19). There are also dental effects in both expansion approaches. Treatment with RME presents dental changes significantly different from patients treated only with fixed appliances (18). These dental effects are expected since the RME appliances are not bone-anchored (13). In this study, skeletal expansion represented 48% of the total transverse increase at the molar level, slightly higher than previously reported (23,24).

The buccal bone thickness decreased significantly at the first molars, while, in general, the palatal bone thickness increased in almost all teeth. This increase in palatal bone thickness was greater at 4 mm from the CEJ, probably due to tooth inclination. Buccal bone loss was observed only in the first molars, possibly because the appliance is supported by bands on these teeth, despite the palatal skeletal anchorage.

Most authors agree that RME produces changes in buccal bone thickness, especially in the region where the appliance is anchored. In this region, there is a reduction of buccal bone and an increase in palatal bone for both RME and MARPE (17,19).

For Haas and Hyrax expanders, Garib *et al*. (14) reported that the buccal bone thickness of the supporting teeth decreased between 0.6 to 0.9 mm while the palatal bone thickness increased from 0.8 to 1.3 mm. As for MARPE, Park *et al*. (6) and Toklu *et al*. (19) observed significant changes in thickness; decreased buccal bone thickness of premolars and first molars ranged from 0.6 to 1.3 mm and palatal bone thickness increased in the first molars and second premolars from 0.3 to 1.3mm.

The buccal inclination of dentoalveolar structures is frequently reported after maxillary expansion (6,14,19). In the present study, a buccal inclination was observed in all teeth, but significant only in the first molars, similar to previous results (6,26). Wilmes, Niekemper and Dreschler (26) reported buccal inclination also in the first premolars. Toklu, Germec-Cakan and Tozlu (19) observed less buccal inclination in the molars, from 2.5° to 3°. These differences between studies may be due to the amount of activation, buccal bone thickness and level or simply methodological differences.

Asscherickx *et al*. (5) used a device anchored only in the bone, without molar support, and also reported molar buccal inclination. This can be explained because part of the change observed in posterior teeth inclination is actually the result of the maxillary rotation when it is divided into two segments during expansion, and not only due to the pure dentoalveolar inclination. According to Cantarella *et al*. (27), for each millimeter of transverse gain in the interzygomatic distance, each half of the maxillary process rotates out 0.6°. This was a limitation of this study, since the CBCT scans were performed only in the maxilla, and the amount of rotation cannot be assessed.

All transverse distances, both dental and skeletal, increased significantly. In the intermolar distance, an increase of approximately 6 mm was observed. Values from 5 to 8.32 mm were observed in the literature (10,23,26). In the premolars, the transverse increase was 3.1 mm, less than the average increase of 6 mm reported in other studies (10,26). In the canines, the increase was 2.8 mm, close to the values previously reported (10,23).

Regarding skeletal measurements, nasal base width increased 2.3 mm. In the literature, the values vary from 1.07 to 2.7 mm (5,6,10,23). The maxillary width increased 2.6 mm in this study, close to values of some studies (6,10). However, other studies have found values above 4mm (5,27). All studies agree that maxillary expansion, regardless of the appliance used, has the effect of a transverse increase in skeletal and dental structures. The variability in the amount of transverse gain is directly related to the amount of activation, which depends on the severity of the maxillary atresia.

The choice of RME or MARPE should consider the age of the patient, the maturation stage of the midpalatal suture and the palate thickness, to increase the chances of success, since the effect in periodontal structures was a significant reduction in the buccal bone thickness and a significant buccal inclination of the first molars, and increases in all dental and skeletal transverse widths. The transverse skeletal increase is approximately half of the transverse dental increase obtained with MARPE.

## Conclusions

After MARPE, there was a significant reduction in the buccal bone thickness of the maxillary first molars and an increase in the palatal bone thickness of all evaluated teeth. The transverse increase was significant for all interdental distances, as well as for nasal base and jugula widths. MARPE caused significant buccal inclination of the maxillary first molars.

The midpalatal suture was opened in a triangular shape, with the smallest increase observed in the nasal base (2.82mm) and the largest increase observed in the intermolar distance (6.37mm). The skeletal expansion, measured in the maxillary width, represented 48% of the total transversal gain measured at the molar level.

The age of the patients was negatively correlated to the transverse skeletal increase. Older patients tended to show a less maxillary transverse skeletal increase. A greater maxillary transverse increase was accompanied by a greater intermolar width increase and also a greater buccal bone loss in the mesiobuccal root of the maxillary first molars.
